# Ration or compassion? Stakeholder perspectives on the introduction of bedaquiline in South Africa

**DOI:** 10.1371/journal.pgph.0004821

**Published:** 2025-07-18

**Authors:** Rene Raad, Graeme Hoddinott, Martin Gorsky, Justin Dixon

**Affiliations:** 1 Department of Global Health and Development, Faculty of Public Health and Policy, London School of Hygiene and Tropical Medicine, London, United Kingdom; 2 Desmond Tutu TB Centre, Department of Paediatrics and Child Health, Faculty of Medicine and Health Sciences, Stellenbosch University, Cape Town, South Africa; 3 School of Public Health, Faculty of Medicine and Health, University of Sydney, Camperdown, New South Wales, Australia; 4 Centre for History in Public Health, Faculty of Public Health and Policy, London School of Hygiene and Tropical Medicine, London, United Kingdom; 5 The Health Research Institute Zimbabwe, Biomedical Research and Training Institute, Harare, Zimbabwe; University of Oxford, UNITED KINGDOM OF GREAT BRITAIN AND NORTHERN IRELAND

## Abstract

Antimicrobial resistance (AMR) is a global health emergency that poses a significant challenge to disease control efforts that rely on antibiotics. Drug-resistant tuberculosis (DR-TB) is a major contributor to global AMR, but its management has historically often remained confined to TB-specific discussions. The emergence of bedaquiline (BDQ), the first novel TB drug in decades, is a moment of potential confluence between AMR and DR-TB. By examining the period between 2012 and 2018, when BDQ was made available for DR-TB in South Africa, this study explores how the introduction of this novel drug foregrounded tensions between antimicrobial access and stewardship in resource-constrained settings. Through qualitative interviews with doctors, policymakers, patients, and activists in the context of DR-TB policy, programming, and care delivery, we explore how these stakeholders balanced the imperative to expand access to this critical new antibiotic and the imperative to ensure its longevity. South Africa, we show, adopted a liberal approach to access to BDQ, grounded in a compassionate care approach that represented a significant shift from the country’s traditional drug rationing aimed at mitigating the spread of DR-TB. We document the numerous obstacles that were faced in enabling compassionate use, as well as the broader implications of South Africa’s liberal BDQ policy both for TB management in South Africa and for global AMR strategies. The BDQ experience suggests that integrating compassionate care into stewardship models can yield positive public health outcomes, challenging some of the foundational assumptions underlying stewardship. In the process, it suggests that a third, balanced strategy is available that explicitly integrates equitable access with robust stewardship to fulfil both immediate and long-term public health goals.

## Introduction

Antimicrobial resistance (AMR) is a threat to global health [1], with a projected 10 million deaths annually by 2050 without intervention [2]. Emergent drug-resistant microbial strains have produced robust discussions around how to manage access to old and new antibiotics responsibly [3]. This “responsible management”, known as antimicrobial stewardship, has assumed a central role in global health and has led to the development of strategies to preserve antibiotics as a class of medicines [1,2,4–6] Strategies to combat AMR have since been developed and refined around several core pillars. The World Health Organization (WHO), for one, has consistently emphasised the importance of awareness, surveillance, reducing infection, stewardship, and research and development [7–9]. The critical need for global collaboration, investment, and research to develop new antimicrobial agents has also featured heavily in the prioritisation of concerns [10–12].

However, despite growing concern, antimicrobial use has increased globally, particularly in low- and middle-income countries (LMICs) [8], contexts within which multiple factors converge to inhibit the prevention and containment of infectious diseases. In parallel, the pace of antibiotic discovery has slowed significantly in the last 60 years. Between 1940 and 1962, over 20 new antibiotic classes were identified [13]. Since then, only three new classes have reached the market. Among these, only one – the class of diarylquinolines – was developed specifically for the treatment of TB [14].

Drug-resistant forms of TB make up one third of the global AMR burden [15], including multidrug-resistant tuberculosis (MDR-TB) – strains resistant to at least isoniazid and rifampicin. Before the approval of bedaquiline (BDQ) in 2014, no new anti-TB compound had entered the market in over forty years [16]. This left patients reliant on lengthy, toxic, and often ineffective regimens with few viable options. The introduction of BDQ addressed a critical gap in treatment options and shifted the landscape of MDR-TB management. BDQ dramatically improved patient experience and treatment outcomes [17], motivating TB stakeholders to advocate for greater access for people with DR-TB. Early access often relied on mechanisms such as “compassionate use”, a framework that allows investigational drugs to be provided outside clinical trials to patients with life-threatening conditions who lack other treatment options [18,19]. However, this development occurred against a backdrop of long-standing challenges in TB programmes, including the emergence of resistance to existing treatments such as rifampicin and isoniazid [20,21]. From this, a bioethical debate emerged about the conflicting concerns of stewardship and treatment prioritisation [22–27].

Stewardship of antimicrobials and access to DR-TB treatment often present conflicting priorities and approaches. Stewardship prioritises antibiotic preservation – including reducing the global reliance on pharmaceutical interventions as a necessary step to maintain their long-term efficacy [28–30]. However, because of the high mortality rate and lack of alternative therapeutic options, TB programming, including care and planning for DR-TB, tends to prioritise expanding access and ensuring the successful implementation of available therapies; in practice, this has often led to antibiotic use that compromises efficacy, particularly in resource-constrained settings where health system infrastructure is weak [21,31]. Despite these competing imperatives, the inclusion of DR-TB in the WHO’s Global AMR Action Plan and other notable reports [1,15,32,33] shows that DR-TB is (at least in policy) a focus of AMR control strategies [34].

Concerns about the excessive use of antibiotics have abounded since their introduction, as illustrated in Podolsky’s [35] and Bud’s [36] histories of antibiotic discovery and distribution, which show how antibiotics became a central and quickly over-depended on part of healthcare service provision. As social scientists and historians of global health have since shown, through initiatives to expand access, antibiotics inadvertently became “quick fixes” to larger public health issues, particularly in LMICs where they were often deployed in part to bridge gaps in under-resourced health systems [37,38]. Often unable to ensure an adequate antibiotic supply through the public sector, in many LMICs loosely-regulated community pharmacies and informal drug shops emerged to fill gaps in formal care, facilitating non-prescription sales and further uncontrolled use of broad-spectrum antibiotics [39–41]. The relative affordability of antibiotics further reinforced this reliance compared to more resource-intensive healthcare interventions, making them an attractive solution especially in LMICs, where financial and infrastructural constraints are pronounced. However, prioritising affordability and scalability often came at the cost of neglecting broader health system strengthening, ultimately leaving many underlying determinants of health inadequately addressed [42,43].

Such deepening antibiotic dependence is captured within the concept of pharamceuticalisation. The focus of a wide body of anthropological and global health scholarship, pharamceuticalisation refers to the process whereby pharmaceutical solutions subsume and replace other, often broader-based interventions in public health policies and practices[cite]. As Jeremy Greene has shown, pharmaceuticalisation in a global context can be traced back to the Essential Medicines movement of the 1970s and 1980s, which aimed to enhance global drug accessibility as a central pillar of primary healthcare [44]. Coupled with the “rational use” movement [45], what constituted an “essential” medicine and ways to avoid misusing it were significantly shaped by the WHO and higher-income countries’ perspectives. While this provided much-needed guidance on which, how, where and when drugs should used, placing antibiotics as the centre of care systems also led to an overemphasis on pharmaceuticals as the critical tool for disease prevention and treatment. This resulted in neglect of the system requirements responsible for their delivery [35].

Although pharmaceuticalisation predates the global health era, it further intensified during this period in the push to ensure wider availability and accessibility of drugs, particularly for HIV, TB, and malaria. A well-rehearsed narrative in critical global health traces how international actors came to increasingly shape healthcare in LMICs, particularly in Africa, which prioritised “vertical” funding and programming, which in turn fragmented service delivery and drew attention away from wider health system strengthening [46,47]. These practices reinforced pharmaceutical-centric approaches and cultivated deepening dependencies on drugs as interventions [48–51]. This reliance highlights the grave risk to health system efficiency, in the context of emergent drug resistance and loss of antibiotic efficacy [38]. With BDQ being one of the few new anti-TB drugs to become available in decades, a significant tension emerges between global health programming’s entrenched logic of ever-expanding access to pharmaceuticals through verticalised approaches, and the emerging imperatives of stewardship in the context of the global AMR emergency – making our analysis particularly pertinent to understanding how these debates have played out in practice. Unlike most broad-spectrum antibiotics, BDQ remains under the tight oversight of national TB programmes and is typically dispensed only through specialist DR-TB services. This exceptional status epitomises the very access–stewardship conflict under review [52,53].

South Africa has the world’s third-largest population of people treated for DR-TB [17,21,54]. As of 2024, MDR-TB accounted for 3.1% of the total TB incidence in the country [55]. Clinical trials in 2012 revealed BDQ to be an effective cure for extensively drug-resistant tuberculosis (XDR-TB) and other resistant strains, with minimal adverse effects. By 2014, South Africa recommended BDQ for treatment for MDR-TB and XDR-TB, prior to the WHO guidance. This prompted international debate about how BDQ would be stewarded responsibly [22–26]. South Africa’s experience with creating access to BDQ is a useful example through which to see how these potentially competing discourses of pharmaceuticalised global health and AMR rationing have played out in a particular setting and programme context.

Our background research involved a scoping review of the evolution of TB containment and control at the intersection of pharmaceuticalisation and drug resistance in South Africa [56]. It showed that the history of TB control in South Africa is one of deepening reliance on pharmaceutical solutions characterised by recurring cycles of drug discovery, optimism, expansion of access, and mounting resistance, culminating in the most recent introduction, optimism, and contestations around BDQ. Building on this formative research, this article provides an in-depth qualitative account of the introduction of BDQ from the perspective of those working within the context of DR-TB control in South Africa. It aims to illustrate how tensions between AMR stewardship and DR-TB treatment priorities played out during BDQ’s initial roll-out and, in doing so, contributes to a deeper understanding of the complexities of balancing these two demands. We do this by (a) contextualising DR-TB care before BDQ was available, and (b) describing key stakeholder perspectives on BDQ access when it first became available and conclude by (c) reflecting on lessons for navigating tensions between AMR stewardship and demand for novel antibiotics. To ensure our analysis centres on the experiences of the TB programme, we specifically gathered insights from stakeholders who are directly involved in DR-TB care, and in the creation and implementation of TB programmes. Our focus was to capture detailed perspectives from those who have a direct role in the administration and policymaking of BDQ in South Africa.

## Methodology

This study used an interview-based qualitative design. It was conducted both in South Africa and, when COVID-19-related lockdowns did not allow for travel and face-to-face research, virtually. Interviews were conducted between November 2021 and December 2023. We sought to include participants who had worked in TB and DR-TB treatment, policy, or advocacy in South Africa over the past 25 years, and we aimed in particular to include those with roles in the introduction of or wider debates around BDQ. Participants were recruited through snowball sampling [57], a technique appropriate when participants needed for the research have experience or traits that are highly specific, as in this context [57]. This enabled us to expand the web of contacts and enquiry and allowed for the identification of individuals whose work intersected within shared contexts, such as national TB programmes, advocacy networks, and healthcare delivery settings.

A semi-structured discussion guide, developed by the research team and informed by the study’s objectives, explored four primary areas: (1) roles and experiences in TB control, (2) the history and introduction of BDQ, (3) the balance between antimicrobial stewardship and treatment access, and (4) the implications of potential loss of BDQ to resistance. While the guide provided a consistent structure, it was then tailored to individual participants’ expertise and roles. For instance, questions for policymakers focused on guideline development, while those for clinicians emphasised implementation challenges and patient outcomes. Interviews lasted approximately 45–60 minutes and were facilitated by a postgraduate anthropologist (RR). Interviews were audio-recorded, and field notes were kept to supplement the analysis.

The key stakeholders comprised eight doctors, six activists, five policymakers, and four TB programme managers. All participants had multiple years’ experience in the roles they held, with close first-hand experiences of DR-TB. Of the 23 participants, three were also TB survivors. Three participants withdrew from participation following data collection.

The data were analysed by using thematic analysis to identify and interpret patterns of meaning within the qualitative data, allowing for an in-depth exploration of the various perspectives on DR-TB management and policy implementation. Braun and Clarke’s six-phase framework was followed [58]. First, all interviews were transcribed verbatim, and the primary researcher (RR) reviewed the transcripts alongside detailed field notes to ensure immersion in the data. Initial codes were generated using an open coding approach, assigning descriptive labels to data excerpts that aligned with the study objectives. Once initial codes were developed, they were grouped into themes based on relevance to the research questions and the overarching study objectives.

### Ethical considerations

The study was reviewed and approved by the University of Stellenbosch’s Human Research Ethics Council (HREC) [REF: S21/03/065] and the London School of Hygiene and Tropical Medicine’s Research Ethics Board [REF: 25353–1]. All methods were carried out in accordance with relevant guidelines and regulations. All participants provided written informed consent to participation and for publication of anonymised findings from the collected data.

## Results

### DR-TB care in South Africa on the arrival of BDQ

Historically, treatment of DR-TB was characterised by complexity, protracted treatment regimens, and limited therapeutic options [59]. Prior to the introduction of advanced diagnostic methods, such as GeneXpert, in 2011, drug sensitivity monitoring was limited and relied heavily on patient history or treatment outcomes. The integration of GeneXpert into national diagnostic protocols by 2013 enabled rapid detection of rifampicin resistance [60]. The roll-out was not without issue, but it did improve the timeliness and accuracy of MDR-TB management relative to those of the strategies before it [61]. Concurrently, from 2012 onwards, BDQ became available through clinical access programmes, initially for limited use in specific contexts, such as compassionate use or research trials. This phased introduction allowed for the evaluation of its safety and efficacy in treating MDR-TB. By 2015, based on these findings and broader availability, BDQ was fully incorporated into South Africa’s national guidelines as a first-line treatment for MDR-TB [62].

Despite these advances, many patients were still treated with MDR-TB protocols, often due to documented exposure to DR-TB, poor outcomes from first-line treatment, or the need for care following a second or subsequent treatment episode. These regimens typically combined first-, second- and third-line treatment options that primarily relied on injectable agents, such as amikacin, kanamycin, and capreomycin, which were associated with severe adverse effects and limited efficacy. In addition to injectables, fluoroquinolones such as ofloxacin and repurposed first-line drugs such as ethambutol and pyrazinamide were often included to broaden the antibacterial spectrum. This combination of older anti-TB drugs, which some of our participants referred to as “salvage drugs”, was costly to the health system [63] and not standardised [64].

Additionally, hospitalisation was required for the combination of these drugs to be tailored to the patient’s needs, as well as to allow the injectables to be administered by health professionals, which perpetuated the bed burden experienced by South African hospitals. For instance, as of 2012, South Africa had approximately 2,500 beds allocated for MDR-TB patients, leaving many patients waitlisted for treatment for months or even years, further exacerbating the spread of DR-TB [65]. The benefits of these injectables were marred by their considerable drawbacks, including ototoxicity and nephrotoxicity. Positive treatment outcomes ranged from 5% to 25% [66]. Fluoroquinolones such as ofloxacin were occasionally featured in conjunction with injectables to expand the spectrum of antibacterial activity. However, while fluoroquinolones represented a step towards more diverse treatment options, access to these drugs remained uneven, particularly in resource-limited settings.

Treatment often lasted for an average of two years or more with devastating adverse effects. If a patient did not respond to the initial regimen, healthcare providers had little to no alternatives – a challenging ordeal that was often further exacerbated by the complexity of treatment adherence. In the absence of a robust and standardised regimen of DR-TB medications, healthcare providers resorted to repurposing older anti-TB drugs, such as those mentioned above, and even medications originally developed for leprosy and other infectious diseases.

One of our participants reported that they experienced extreme weight loss, needing to use a wheelchair, persistent nausea, hair loss, and a change in eye colour. More broadly, patients undergoing treatment for MDR-TB have been known to lose their sight and hearing completely, or suffer from peripheral neuropathy or kidney failure. The adverse effects made the treatment of MDR-TB a specialist issue that required inpatient care and close monitoring. However, insufficient hospital beds left patients waitlisted for years. A doctor we spoke to described how:

[In] the eight subdistricts in Cape Town, maybe you’ll have a thousand patients diagnosed with DR-TB a year, and there are two hundred beds in [the hospital]. And on average, people would stay until they finished their injectable agent, which would be about six months … if you do the maths quickly, you’ll recognise that people are waiting for three years to get into hospital.

The doctor added that, without admission, patients were additionally at risk of their TB advancing, of dying, and of further transmitting the infection within their households and communities.

Health advisors who happened to be patients during this time lamented the challenges they had faced in their efforts to receive treatment. One disclosed that their doctors had begun working outside official guidelines to make medicines accessible to waitlisted patients. They recalled the day their doctor, conflicted by the challenges of the policies, told them they were going to “break the rules”. Bound only by a promise that they would take their medicines, the patient was administered MDR-TB treatment in secret.

Cases of doctors informally administering medicines appeared in our data numerous times – particularly after BDQ became available for compassionate use. Of the doctors we spoke to, most reflected on how “really, really depressing” it had been to treat TB during this time and how conflicted they had felt when facing their suffering patients. Notably, one doctor said “focus began to shift toward palliative care”, and “controversy around keeping patients in hospital” had emerged when beds were in desperate demand.

The introduction of BDQ into the landscape of DR-TB care provided optimism for both providers and patients. It promised shorter treatment durations, reduced toxicity, and dramatically improved treatment outcomes. However, despite positive treatment outcomes, BDQ was available only to a limited group of patients, as it awaited larger clinical trials. This meant that not all MDR-TB patients could access the treatment option. One doctor stated that the benefits of BDQ were:

So obvious that in the end [when] most civil society groups and clinicians and policymakers … came together, [they] were just like, “This is unethical”. You can no longer sit in on the front line of someone and say “This is your regimen. I’m going to give you an injection, which will make you go deaf probably and you have a 50% chance of survival. Whereas we actually do have access to this other wonder drug, which we aren’t going to give to you because you are not sick enough”.

### Expansion and contestations over BDQ use

In 2007, activists from Médecins Sans Frontières (MSF), the Treatment Action Campaign (TAC), the Treatment Action Group (TAG), the European AIDS Treatment Group (EATG), and a representative of the Stop TB Partnership came together to request compassionate access to TMC207, which later became known as BDQ [66]. Compassionate use would allow patients to access the drug before clinical trials were completed, offering respite to individuals who had exhausted all other treatment options. One healthcare provider shared their perspective on this critical turning point: “We heard about BDQ [through] the TB conferences – the international ones … It had got to the penultimate stage of drug trials, and when those trial results were published, it was truly unbelievable.”

Although compassionate use programmes, also known as special access initiatives, had historically been implemented in the contexts of cancer and HIV/AIDS, the WHO extended the application of this principle to DR-TB in light of the poor treatment outcomes and the clinical development of BDQ [67].

However, apprehensions about the stewarded use of BDQ persisted among healthcare providers, highlighting a contentious debate over its regulation and administration. These concerns were not just clinical but also ethical, reflecting a broader uncertainty within the global health community about how best to balance the immediate needs of patients with the long-term goals of antimicrobial stewardship. It highlights the challenging nature of healthcare decision-making, particularly in the face of new and potentially lifesaving treatments. The experiences of providers during these early days of BDQ access demonstrate the many dynamics that shape such decisions and reveal the ethical dilemmas and practical challenges, such as issues of accessibility and responsible drug stewardship, that were encountered by the stakeholders involved in the roll-out.

Healthcare providers recognised that compassionate use could provide hope to patients who had no other treatment options. While the idea of compassionate use was transformative, its implementation was challenging. In 2011, Janssen Pharmaceuticals, the pharmaceutical company that developed BDQ, created a compassionate use programme that would provide BDQ, free of charge, to a limited number of patients before the final approval and marketing of the drug. To qualify, these patients had to have exhausted their treatment options. Multiple countries joined the programme; however, out of caution, the South African Medicines Control Council (MCC) revoked permission for its citizens to participate – until further safety data had been provided [68].

Between 2011 and 2012, more attempts were made to create access to BDQ for compassionate use. MSF quickly formed a trial and requested access to BDQ through clinical access. A collaborative proposal was developed by MSF, Janssen Pharmaceuticals, the Southern African HIV Clinicians Society, and the National Department of Health (NDoH), proposing a Clinical Access Programme protocol. The protocol aimed to provide BDQ at select, well-equipped sites renowned for their extensive experience in managing DR-TB. This, along with several iterations after that, was rejected by the MCC. In December 2012, it was approved, and in 2013, the Bedaquiline Clinical Access Programme (BCAP) was rolled out in South Africa to enrol 3,000 patients [69,70].

In 2015, BDQ access was expanded to include patients experiencing adverse reactions to the standard treatment regimens [71]. To facilitate this, healthcare providers were able to apply for approval to prescribe BDQ through a standardised application process. However, despite the changes, healthcare providers still felt constrained by the guidelines governing it. The bureaucratic process was cumbersome, requiring committee approvals and extensive paperwork. A healthcare provider described the process:

I mean, the paperwork was phenomenal … you’d identify your patient and then three months later you would be able to give them bedaquiline … and you[re] thinking “My patient’s going to be dead by the time they get this drug”.

To address the limited eligibility criteria, some providers sought creative solutions. For instance, one participant described how they found a workaround within the inclusion criteria:

One of the inclusion criteria was that you had toxicity to the injectable and so one of the toxicities is hearing loss … so when you were calling your patients to come in, you would whisper their name and when they wouldn’t come then you’d be like “hearing loss” [to meet the criteria].

The doctor emphasised that they could easily live with themselves for doing this, saying “How are you going to give this person this awful drug when there’s something else available?”

Working around the guidelines was not limited to practitioners. A health advisor we spoke to had also guided patients on getting doctors to prescribe BDQ by telling doctors they were struggling to hear. The anecdotes highlight the urgency and determination among healthcare providers to get access to BDQ for their patients.

In 2014, South Africa made the bold decision to provide BDQ to all MDR-TB patients as a first-line treatment option, which was shorter and injection-free. This decision marked a departure from earlier protocols that relied heavily on injectable drugs with severe side effects.

… the South African Department of Health made this huge leap, which was unprecedented across the world really, where they said … “Not only are we going to give bedaquiline to everyone, but we’re going to give it within a shorter, nine-month regimen. So, we are not going to treat for two years anymore … we’re going to treat with bedaquiline, without an injection, for nine months”.

The shift was driven by several factors, including the integration of GeneXpert into South Africa’s diagnostic protocols, which enabled the rapid identification of drug resistance [72,73]; the growing body of evidence from global clinical trials demonstrating BDQ’s efficacy [74]; and the intense advocacy efforts by civil society groups and healthcare providers, as described above.

However, South Africa’s decision was not without criticism. As one doctor described, “South Africa [was] ridiculed”; they recalled how the consensus had been “You are crazy. This has never been tested on such a level. You can’t do this as a national policy”. The sceptical response to South Africa’s initiative highlighted the concern about the untested nature of the approach as well as the risk that it might lead to drug resistance. “You’re just absolutely going to destroy it; you’re going to get BDQ resistance in TB”, the doctor recalled being told.

### Linezolid–BDQ combination therapy: Mitigating drug and political resistance

In recognition of the potential for the development of resistance, mitigation plans were put in place prior to BDQ’s approval by a dedicated stewardship team at the NDoH and MSF to preserve the effectiveness of BDQ. In 2012, MSF initiated access to linezolid due to its effectiveness in treating DR-TB and its ability to bolster protection for BDQ’s efficacy [75]. By introducing a second drug, they aimed to slow down resistance development. This combination therapy approach was founded on the principle that using two drugs simultaneously would reduce the likelihood of resistance development. However, despite these proactive efforts, “Major difficulties [were] experienced in obtaining the companion drugs” [70].

Between 2011 and 2014, MSF and later the NDoH engaged in an enormous undertaking to create access to linezolid. Starting in August 2011, MSF used linezolid in their treatment regimens in Khayelitsha. Facing the high costs of the drug, MSF requested a price reduction in 2012 but received no response. In 2013, it turned to a UK-registered generic version, significantly cheaper than the branded one, but faced regulatory hurdles for its use in South Africa. MSF’s application to South Africa’s MCC in December 2013 for special permission to use the generic drug was initially denied due to affordability not being deemed a suitable consideration for granting such approvals. This led MSF to file an appeal in March 2014 and eventually resort to litigation in June 2014 [75].

“We were having to smuggle it in…” one of our participants said before going on to express their frustration, and at times disgust, with pharmaceutical companies: “Those of us who were introducing [BDQ] were not ignorant to [the risk of developing resistance]. We knew that that was a fear. So, we knew that we needed to introduce it along with another novel drug.” Yet, once again, they were met with the significant challenge of accessing these critical drugs, highlighting an ongoing barrier to providing comprehensive and safe patient care and the responsible administration of these medications.

In 2014, the South African media reported on what became known as the “Pharmagate” scandal. This revelation centred on the efforts of major pharmaceutical companies to hinder policy reforms that threatened their patent monopolies. The South African newspaper the *Mail & Guardian* reported on the scandal, which had ignited a wave of anger among South African health officials, most notably the Minister of Health, Aaron Motsoaledi, who vehemently criticised the pharmaceutical companies’ actions as detrimental to public health [76]. Motsoaledi called the restrictions a “satanic plot” from “Big Pharma” and likened it to “genocide” [76]. In the wake of the scandal, political parties across South Africa pledged to prioritise the finalisation of a pro-public-health intellectual property policy, which would prevent protracted legal battles over access to generic versions of lifesaving drugs, as in the case of generic linezolid [75,76]. The policy was approved in 2018 [77].

In the case of linezolid, the situation was resolved in June 2014 when the MCC granted MSF approval to import the generic version following out-of-court negotiations and additional quality assurances provided by MSF. Despite the challenges of procuring linezolid, its availability, alongside BDQ, transformed how South Africa approached DR-TB treatment. Instead of saving these drugs as a last resort, providers were providing the most effective regimens upfront to prevent resistance development. As one participant stated, “You have one chance. Right up front, you get the best regimen you can … and if that doesn’t work, then you go to all the salvage stuff … We were no longer saving drugs”. They added “We were saying let’s use everything that we can … the best that we have right upfront to prevent that acquisition of resistance”.

However, this proactive yet risky approach did not come without its controversies and challenges. The decision to administer these potent drugs as first-line treatments led to challenging times for some of those at the forefront of the effort. One participant shared that this period “was quite painful”, recalling a time when leaders in the South African government would quietly call for their resignation, due to the high stakes of their strategic choices; this highlighted the tensions that arose during the act of balancing immediate patient needs and long-term public health objectives, a central dilemma in AMR stewardship.

### Power dynamics and coloniality in global stewardship

The shift from keeping novel drugs as a last resort to using them routinely in MDR-TB treatment represented a significant change in the philosophy of treating DR-TB in South Africa. The work put into BDQ paved the way for other drugs to become more easily accessible. “It was easier to implement it”, a doctor said. “It was easier to roll it out because we just used the existing processes in ways that we’d rolled out BDQ. We just did the same for [other drugs].”

When asked whether concern for emergent resistance was warranted, healthcare providers acknowledged the inevitability of resistance development. As one participant put it, “We’re already seeing BDQ resistance come through … We need to have this constant supply, this constant pipeline of new drugs coming through to keep getting the next resistant strain”. They added “We have [destroyed it] now … but I mean, that’s the thing … it was always going to happen, right? That’s just the evolution of TB”. Another said:

You hear this language all the time, “We want to protect the drug”, and there were a few of us who were saying “What about protecting the people who are sick? What does it mean to protect the drug?” So, in the sense of, if we use it the right way, they’ll never be resistant, which is a fallacy, I mean there is always resistance, it’s evolution right? It’s just the way bacteria works, and TB is no different in that regard.

They later recalled a gathering where they were “yelled at” while talking about creating access to BDQ. Someone they described as “notable” and “prominent”, from a higher-income country setting, stood up and accused them of “wasting this drug”. “Why is it wasting a drug to treat sick people?” they had asked, visibly frustrated by the assertion.

[M]uch of this conversation [is] really laced with a colonial attitude … people would say “We need to protect BDQ” and I would say “What do you mean by that?” … You’d hear it parroted everywhere you went: “We have to protect the drug” … What does that mean? Because what’s inherent in that is that somebody is going to misuse it, and inherent in that is that [there are] people who should get it who are good and people who shouldn’t, who are bad. People would also say “We need to protect the drug in case we need it in the future”. [I would say] “But we need it right now” … how bad does it have to get…? What is that about?

When asked what they believed the implicit message was, the doctor responded:

The underlying conversation was [always] “Protect the drug in case we need it in wealthy countries for white people in the future” … I [would ask] “Do you think my patients in South Africa don’t deserve this? Do you think the man sitting in [Cape Town] … shouldn’t he get bedaquiline because some unnamed person in the US might?” … There is a lot of colonialism and there’s a lot of racism that drives these conversations and this contempt that people have towards people with TB … like they’re going to misuse it, or they’re going to waste it … when that stuff is cloaked in this mantle in public health responsibility, it becomes very dangerous.

## Discussion

From the perspective of those working within the TB programme in South Africa, stewardship of the new “wonder drug” BDQ became synonymous with access denial. This created an unhelpful polarisation and politicisation of “strawman” camps between opposing viewpoints: on the one hand, there were fears of “wasting” or “ruining” one of the first new antibiotics through the pipeline in decades, and on the other, concerns about enacting a thinly veiled colonial agenda to preserve new drugs for patients in the Global North. This polarisation is particularly relevant in South Africa, where structural challenges intersect with the need for equitable access to lifesaving drugs like BDQ. In this challenging landscape, traditional notions of stewardship became untenable. Even when there were hard policy barriers in place constraining access, providers and patients found themselves bypassing regulations, driven by an ethical imperative to prioritise care.

Global health ethics scholarship has framed this dilemma as a tension between access (the moral duty to relieve present suffering) and excess (the risk that unrestrained consumption accelerates collective harm) [27]. Situating BDQ along this “access–excess” spectrum recasts South Africa’s policy choice somewhat as an ethics experiment privileging distributive justice for today’s patients over a precautionary, future-oriented stewardship ideal. Commentators argue that such trade-offs are inevitable and should be debated openly by a wide range of stakeholders, rather than decided by a small group of technical experts. [27].

These issues are especially acute in South Africa, a country with an entrenched history of poverty, inequality, and racial discrimination. These structural challenges have not only promoted the spread of TB but also compounded the difficulties in treating drug-resistant forms of the disease [78]. Participants in this research powerfully described the daily struggles of seeking to secure medicines and care before the introduction of BDQ, particularly in the throes of the MDR-TB and XDR-TB outbreaks. Doctors consistently emphasised the sense of helplessness they experienced when confronted with abysmal treatment outcomes for their patients. Making it unsurprising that when BDQ emerged as a potential solution, the prevailing sentiment was one of excitement.

Despite the consensus on the inevitability of resistance to BDQ and subsequent novel treatments, participants did not waver in their belief in prioritising access to these drugs. Importantly, they had also carefully considered the argument that novel treatments should be restricted or limited in use; queried whom the preservation of drugs was truly intended to serve; and vehemently fought – often at personal risk – to introduce linezolid to mitigate the potential for the development of resistance. What they were concerned about, however, was the way global stewardship and “drug preservation” discourses implicitly positioned those in low-resource settings as “abusing” antibiotics and suggested that a notion of ‘deserving’ (and thus undeserving) was subtly slipping into these questions, which our participants felt required more interrogation.

The notion of individual behaviour, rather than systemic and structural factors, occupying the centre stage in public health discourse is intimately familiar to social scientists working in the context of TB. Paul Farmer [79], for instance, long argued that the emphasis on adherence and the consequences of defaulting draws attention away from the structural violence that renders patients susceptible to TB. This critique resonates with South Africa’s experience, where the emphasis on individual behaviour often overlooks systemic inequities that influence health outcomes. This emphasis, which was exported around the world through the Directly Observed Tuberculosis strategy (DOTS) model, neglected to consider the impact of structural violence on patients’ ability to attend clinics and placed the responsibility for navigating it on the shoulders of vulnerable communities, emphasising a disconnect between policy and practice when implemented in low-resource settings.

The impacts of these discourses have been well documented, including in South Africa [80–82]. More recently, social scientists working in the context of AMR have observed that much of the emphasis of contemporary stewardship has tended to be placed on the “irrational” behaviour of patients and the restriction and correction of use to protect drug efficacy [83–86]. Building on these critiques, Chandler, Hutchinson, and Hutchison [87], reviewing the collective insights of social scientists within the field of antibiotic use and resistance, have suggested that while policy-level “talk” about protecting vulnerable medicines expands, considerations of protecting vulnerable people have receded – a trend that is particularly concerning for LMICs, as most discussions have been driven by European actors [87] creating a top-down approach to solving a problem that sits predominantly in LMIC contexts.

With BDQ being the first novel TB drug in 40 years, the stakes in making it a first-line drug in contexts such as South Africa were especially high. South Africa was perhaps unsurprisingly placed under considerable scrutiny as to whether the adoption of BDQ was a safe and sustainable strategy [23,88]. From the perspective of our participants, the dominant position within the global TB community was that BDQ be reserved for patients with limited treatment options – an opinion so resounding that it fed into the early WHO guidance [89,90]. This caution was adopted by countries such as India, which approached its roll-out more conservatively. BDQ use in India was initially restricted to specialised centres and governed by strict regulatory frameworks under the Conditional Access Programme (CAP), introduced in 2016 [91]. While this approach aimed to mitigate potential adverse effects and the emergence of drug resistance, it limited access for many patients with MDR-TB, leaving them reliant on older, less effective regimens, and excluded others who could have benefited from its use [92,93]. These barriers likely reduced the overall pace of progress – a result South Africa actively wanted to avoid.

Since then, South Africa has monitored its real-world impact, an approach that allowed the country’s lessons to be shared and ultimately fed into WHO guidance in 2018, which recommended the drug for most patients being treated for MDR-TB around the world [17,94]. The argument for compassion over the rationing of BDQ [23] resonates with arguments to put care for people back into a conversation increasingly framed around care for medicines [87,95]. South Africa’s decision to make BDQ widely available reflects a compassionate approach that has fortunately yielded positive outcomes in the fight against DR-TB. South Africa’s success in championing compassionate use and influencing WHO policy despite global AMR imperatives raises critical questions about pushing back against restrictive drug rationing for the “greater good”.

However, the concerns around the risks associated with introducing these drugs into environments that have historically struggled to ensure adherence are not entirely unwarranted. In 2024, significant signs of resistance to BDQ have rapidly developed [96]. This suggests that an old pattern may be about to repeat, as we have recently observed through our aforementioned scoping review of South Africa’s TB programme [56]. Moreover, the evolving pharmaceuticalisation of South Africa’s TB programme over time has significantly increased reliance on these drugs. This is a trend that, as many critical global health scholars have observed [43,97], was accelerated during the global health era due to the intensified distribution of drugs for TB, HIV, and malaria.

This context gains urgency as South Africa now contends with the complexities introduced by the BPAL(M) regimen, which incorporates BDQ alongside other novel and much-relied-upon drugs: pretomanid, linezolid, and moxifloxacin. This combination offers shorter, less toxic treatments and have been embraced as BDQ and delamanid were before them [94]. However, affordability, access, and resistance management remain critical challenges. South Africa’s experience with BDQ provides valuable lessons for responsibly scaling up BPAL(M), especially in resource-limited settings facing persistent structural barriers.

South Africa’s experience emphasises the increased urgency of the need for stewardship approaches that balance pharmaceutical and non-pharmaceutical interventions. AMR stewardship emphasises the need to prevent excessive use of antibiotics, while ensuring access. However, South Africa’s efforts to make BDQ accessible highlight the challenges of implementing stewardship principles within LMIC contexts, which are historically reliant on pharmaceutical interventions to manage health challenges. This dependency is reflected in the cyclical reliance on quick pharmaceutical fixes driven by entrenched processes of pharmaceuticalisation. Given this, we argue that the focus of AMR stewardship efforts needs to shift from solely regulating the use of drugs to additionally addressing the underlying determinants of health that render individuals vulnerable to infection. For example, discussions around compassionate use were framed almost exclusively in terms of drug availability and immediate patient care, rather than as part of a larger effort to reduce long-term pharmaceutical dependence.

Tending to social and infrastructural factors at the same time as promoting rational drug use will be crucial if stewardship is ever to succeed in LMICs. However, with South Africa’s ongoing struggles politically, socially, and economically, it remains challenging to achieve a universal healthcare system. Acknowledging these constraints, it becomes imperative to strengthen health systems, invest in improving living conditions, and incorporate more comprehensive social care in health system approaches. Such steps are imperative not only for the effectiveness of AMR stewardship efforts but also to break the cycle of dependency on pharmaceuticals as a solution to global health emergencies.

### Strengths and limitations of the study

The strengths of our methods are in our approach to gathering diverse perspectives from a wide range of stakeholders involved in DR-TB clinical care and programming in a high-burden, low-resource setting. This holistic gathering of data allowed us to capture a comprehensive view of the challenges and expectations surrounding the introduction of BDQ. However, the particularity of participants’ perspectives to South Africa is a potential limitation on the transferability of the findings.

Additionally, despite including a broad range of stakeholders related to DR-TB, our data and analysis do not include a “pro-stewardship/reduce access” perspective. Only one participant expressed a dissenting view, and they requested that it not be included, citing concerns about contributing to “polemics”. When asked about the absence of dissenting voices more broadly, some participants speculated that initial concerns about liberalised BDQ access had diminished over time, as the policy’s success had alleviated fears. Others suggested that the small, interconnected nature of South Africa’s TB care community might discourage open expression of dissenting views due to potential reputational risks or the desire to maintain social cohesion; this is something we did experience when asking participants for referrals, which they most often declined to give.

These factors likely contributed to the limited representation of opposing perspectives in our findings. This omission may have led to an underrepresentation in the analysis of concerns and strategies pertinent to AMR stewardship. While this is a limitation, we argue that it also provides further insight into the broader consensus within South Africa’s high-burden context, where the urgency of addressing DR-TB and reducing mortality often takes precedence over long-term stewardship goals. Nevertheless, future research should aim to incorporate these perspectives to present a more comprehensive understanding of the challenges and potential solutions encountered in managing DR-TB within the broader AMR framework.

## Conclusion

This study highlighted how the effort to expand access to BDQ in South Africa represented a significant shift from the traditional stewardship approaches that have often prioritised preserving antibiotics for future use over meeting immediate patient needs. What we learn is that if those working in LMIC settings are forced to choose between preserving drugs or expanding access, they will inevitably choose access. Further to that, any attempt to enforce stewardship in such settings will likely be thwarted or worked around through community-, provider-, and patient-level collaborations.

What is needed, then, is a conceptual reframing, such that stewardship efforts are not framed by the unhelpful dichotomy of preserving drugs *versus* preserving life. Instead, there should be a move towards working with affected communities of antimicrobial users to collaborate on strategies that address both immediate patient needs and long-term public health goals.

South Africa’s bold and compassionate approach demonstrates how those working within high-burden settings can prioritise patient needs while still influencing global policy, as seen in its impact on WHO guidance. Effective implementation of AMR stewardship in high-burden, lower-resourced settings must be about building public health capacity and addressing underlying social determinants so that we can preserve life with the effective use of drugs.

## Supporting information

S1 Checklist (DOCX)

## References

[1] WHO. Global Action Plan on Antimicrobial Resistance. Microbe Magazine. 2015. doi: 10.1128/microbe.10.354.1

[2] World Bank Group. Antimicrobial Resistance (AMR). In: World Bank Brief [Internet]. 2021 [cited 17 Nov 2021]. Available from: https://www.worldbank.org/en/topic/health/brief/antimicrobial-resistance-amr

[3] Rosenblatt-Farrell N. The landscape of antibiotic resistance. Environ Health Perspect. 2009;117(6):A244-50. doi: 10.1289/ehp.117-a244 19590668 PMC2702430

[4] WHO. Antibiotic resistance. World Health Organization; 2018.

[5] WHO. Global strategy and targets for tuberculosis prevention, care and control after 2015. 2013.

[6] World Bank Group. Pulling Together to Beat Superbugs Knowledge and Implementation Gaps in Addressing Antimicrobial Resistance. The World Bank Report. 2019. doi: 10.1596/32552

[7] Tacconelli E, Carrara E, Savoldi A, Harbarth S, Mendelson M, Monnet DL, et al. Discovery, research, and development of new antibiotics: the WHO priority list of antibiotic-resistant bacteria and tuberculosis. Lancet Infect Dis. 2018;18(3):318–27. doi: 10.1016/S1473-3099(17)30753-3 29276051

[8] WHO. Report on Surveillance of Antibiotic Consumption. WHO; 2018. p. 128. Available from: https://apps.who.int/iris/bitstream/handle/10665/277359/9789241514880-eng.pdf

[9] WHO. Global Framework for Development & Stewardship to Combat Antimicrobial Resistance. 2018.

[10] Howard SJ, Hopwood S, Davies SC. Antimicrobial resistance: a global challenge. Sci Transl Med. 2014;6(236):236ed10. doi: 10.1126/scitranslmed.3009315 24828073

[11] van Hengel AJ, Marin L. Research, Innovation, and Policy: An Alliance Combating Antimicrobial Resistance. Trends Microbiol. 2019;27(4):287–9. doi: 10.1016/j.tim.2018.12.005 30638776

[12] Spruijt P, Petersen AC. Multilevel governance of antimicrobial resistance risks: a literature review. J Risk Res. 2020;25(8):945–58. doi: 10.1080/13669877.2020.1779784

[13] Coates ARM, Halls G, Hu Y. Novel classes of antibiotics or more of the same? Br J Pharmacol. 2011;163(1):184–94. doi: 10.1111/j.1476-5381.2011.01250.x 21323894 PMC3085877

[14] Sulis G, Sayood S, Gandra S. Antimicrobial resistance in low- and middle-income countries: current status and future directions. Expert Rev Anti Infect Ther. 2022;20(2):147–60. doi: 10.1080/14787210.2021.1951705 34225545

[15] The Review on Antimicrobial Resistance (Chaired by Jim O’Neill). Tackling Drug-Resistant Infections Globally: Final Report and Recommendations. 2016. p. 1–80.

[16] Masini T, Hauser J, Kuwana R, Nhat Linh N, Jaramillo E. Will regulatory issues continue to be a major barrier to access to bedaquiline and delamanid? Eur Respir J. 2018;51(3):1702480. doi: 10.1183/13993003.02480-2017 29567722

[17] WHO. Consolidated guidelines on tuberculosis. Module 4: Drug-resistant tuberculosis treatment. WHO; 2020.

[18] Rahbari M, Rahbari NN. Compassionate use of medicinal products in Europe: current status and perspectives. Bull World Health Organ. 2011;89(3):163. doi: 10.2471/BLT.10.085712 21379408 PMC3044254

[19] Borysowski J, Ehni H-J, Górski A. Ethics review in compassionate use. BMC Med. 2017;15(1):136. doi: 10.1186/s12916-017-0910-9 28735571 PMC5523146

[20] Mvelase NR, Balakrishna Y, Lutchminarain K, Mlisana K. Evolving rifampicin and isoniazid mono-resistance in a high multidrug-resistant and extensively drug-resistant tuberculosis region: a retrospective data analysis. BMJ Open. 2019;9(11):e031663. doi: 10.1136/bmjopen-2019-031663 31699736 PMC6858147

[21] Cox Helen, Dickson-Hall L, Jassat W, Moshabela M, Kielman K, Grant A, et al. Drug-resistant tuberculosis in South Africa: history, progress and opportunities for achieving universal access to diagnosis and effective treatment. S Afr Health Rev. 2017;20: 157–68. Available from: https://hdl.handle.net/10520/EJC-c84603e5b

[22] Dheda K, Esmail A, Limberis J, Maartens G. Selected questions and controversies about bedaquiline: a view from the field. Int J Tuberc Lung Dis. 2016;20(12):24–32. doi: 10.5588/ijtld.16.0065 28240569

[23] Furin J. The potential perils of a drug protection framework in tuberculosis. Lancet Infect Dis. 2022;22: 432–33. doi: 10.1016/S1473-3099(21)00681-2 34780707

[24] Kunkel A, Furin J, Cohen T. Population implications of the use of bedaquiline in people with extensively drug-resistant tuberculosis: are fears of resistance justified? Lancet Infect Dis. 2017;17(12):e429–33. doi: 10.1016/S1473-3099(17)30299-2 28533094

[25] Bloemberg GV, Gagneux S, Bottger EC. Acquired Resistance to Bedaquiline and Delamanid in Therapy for Tuberculosis. N Engl J Med. 2015;373: 2–4. doi: 10.1056/NEJMc1505196.Acquired PMC4681277 26559594

[26] Jain A. TB institute warns against use of new drug. The Hindu Newspaper. 9 Jan 2017.

[27] Pokharel S, Adhikari B, Johnson T, Cheah PY. Interventions to address antimicrobial resistance: an ethical analysis of key tensions and how they apply in low- income and middle-income countries. BMJ Glob Health. 2024;9(4):e012874. doi: 10.1136/bmjgh-2023-012874 38569658 PMC11002359

[28] Padayatchi N, Mahomed S, Loveday M, Naidoo K. Antibiotic stewardship for drug resistant tuberculosis. Expert Opin Pharmacother. 2016;17(15):1981–3. doi: 10.1080/14656566.2016.1225724 27550243

[29] Rogers Van Katwyk S, Giubilini A, Kirchhelle C, Weldon I, Harrison M, McLean A, et al. Exploring Models for an International Legal Agreement on the Global Antimicrobial Commons: Lessons from Climate Agreements. Health Care Anal. 2023;31(1):25–46. doi: 10.1007/s10728-019-00389-3 31965398 PMC10042908

[30] Årdal C, Outterson K, Hoffman SJ, Ghafur A, Sharland M, Ranganathan N, et al. International cooperation to improve access to and sustain effectiveness of antimicrobials. Lancet. 2016;387(10015):296–307. doi: 10.1016/S0140-6736(15)00470-5 26603920

[31] Glatthaar E. Tuberculosis control in South Africa: “Where have we gone wrong?” and “A look into the future.” South African Med J. 1982;62:36–41. doi: 10.1542/peds.66.2.326a 7135105

[32] World Bank. Stopping the Grand Pandemic: A Framework for Action Addressing Antimicrobial Resistance through World Bank Operations Previous STEP-BAC. 2024.

[33] World Bank. Drug-resistant infections: a threat to our economic future. Washington, DC; 2017 Mar. Available from: www.worldbank.org

[34] Stop TB Partnership. The Global Plan to End TB 2023-2030. 2023.

[35] Podolsky SH. The Antibiotic Era: Reform, Resistance, and the Pursuit of a Rational Therapeutics. Baltimore, Maryland: Johns Hopkins University Press; 2015.

[36] Pen RB. Penicillin: triumph and tragedy. London: Oxford University Press; 2007.

[37] Chandler CIR. Current accounts of antimicrobial resistance: stabilisation, individualisation and antibiotics as infrastructure. Palgrave Commun. 2019;5(1):53. doi: 10.1057/s41599-019-0263-4 31157116 PMC6542671

[38] Bowker GC, Star SL. Sorting Things Out: Classification and its Consequences. Am J Nurs. 1999. doi: 10.2307/3421475

[39] Auta A, Hadi MA, Oga E, Adewuyi EO, Abdu-Aguye SN, Adeloye D, et al. Global access to antibiotics without prescription in community pharmacies: A systematic review and meta-analysis. J Infect. 2019;78(1):8–18. doi: 10.1016/j.jinf.2018.07.001 29981773

[40] Ocan M, Obuku EA, Bwanga F, Akena D, Richard S, Ogwal-Okeng J, et al. Household antimicrobial self-medication: a systematic review and meta-analysis of the burden, risk factors and outcomes in developing countries. BMC Public Health. 2015;15:742. doi: 10.1186/s12889-015-2109-3 26231758 PMC4522083

[41] Morgan DJ, Okeke IN, Laxminarayan R, Perencevich EN, Weisenberg S. Non-prescription antimicrobial use worldwide: a systematic review. Lancet Infect Dis. 2011;11(9):692–701. doi: 10.1016/S1473-3099(11)70054-8 21659004 PMC3543997

[42] Biehl J. Pharmaceuticalization: AIDS treatment and global health politics. Anthropol Q. 2007;80:1083–126. doi: 10.1353/anq.2007.0056

[43] Packard R. A history of global health: Interventions into the lives of other peoples. Baltimore: Johns Hopkins Press; 2016.

[44] Greene J. Prescribing by Numbers. 2007.

[45] WHO. The rational use of drugs: Report of the conference of experts. Conference of Experts on the Rational Use of Drugs. Nairobi; 1985. Available from: https://iris.who.int/handle/10665/37174

[46] Keugoung B, Macq J, Buve A, Meli J, Criel B. The interface between the national tuberculosis control programme and district hospitals in Cameroon: missed opportunities for strengthening the local health system -a multiple case study. BMC Public Health. 2013;13:265. doi: 10.1186/1471-2458-13-265 23521866 PMC3626530

[47] Marchal B, Cavalli A, Kegels G. Global health actors claim to support health system strengthening: is this reality or rhetoric? PLoS Med. 2009;6(4):e1000059. doi: 10.1371/journal.pmed.1000059 19399158 PMC2667637

[48] Biehl J. Pharmaceuticalization: AIDS treatment and global health politics. Anthropol Quarterly. 2007.

[49] Biehl J. Drugs for all: the future of global AIDS treatment. Med Anthropol. 2008;27(2):99–105. doi: 10.1080/01459740802022777 18464125

[50] Abraham J. Pharmaceuticalization of Society in Context: Theoretical, Empirical and Health Dimensions. Sociology. 2010;44(4):603–22. doi: 10.1177/0038038510369368

[51] Bell SE, Figert AE. Medicalization and pharmaceuticalization at the intersections: Looking backward, sideways and forward. Soc Sci Med. 2012;75(5):775–83. doi: 10.1016/j.socscimed.2012.04.002 22633161

[52] WHO. WHO best-practice statement on the off-label use of bedaquiline and delamanid for the treatment of multidrug-resistant tuberculosis. 2017 [cited 26 May 2025]. Available from: http://apps.who.int/bookorders

[53] Rutta E, Kambili C, Mukadi Y. The Bedaquiline Donation Program: progress and lessons learned after 4 years of implementation. Int J Tuberc Lung Dis. 2020;24(10):1039–45. doi: 10.5588/ijtld.20.0134 33126936

[54] Wood R, Lawn SD, Johnstone-Robertson S, Bekker L-G. Tuberculosis control has failed in South Africa--time to reappraise strategy. S Afr Med J. 2011;101(2):111–4. doi: 10.7196/samj.4587 21678737

[55] WHO. 2024 Global Tuberculosis Report. 2024.

[56] Raad R, Dixon J, Gorsky M, Hoddinott G. Cycles of antibiotic use and emergent antimicrobial resistance in the South African tuberculosis programme (1950-2021): A scoping review and critical reflections on stewardship. Glob Public Health. 2024;19(1):2356623. doi: 10.1080/17441692.2024.2356623 38771831

[57] Russell BH. Nonprobability Sampling and Choosing Informants. In: Research Methods in Anthropology: Qualitative and Quantitative Approaches. 4th ed. AltaMira Press; 2006. p. 186–209.

[58] Braun V, Clarke V. Using thematic analysis in psychology. Qual Res Psychol. 2006;3(2):77–101. doi: 10.1191/1478088706qp063oa

[59] Ausi Y, Santoso P, Sunjaya DK, Barliana MI. Between Curing and Torturing: Burden of Adverse Reaction in Drug-Resistant Tuberculosis Therapy. In: Patient Preference and Adherence. Dove Medical Press Ltd; 2021. p. 2597–607. doi: 10.2147/PPA.S333111 PMC8627322 34848950

[60] National Health Laboratory Service (NHLS). GeneXpert Progress Report December 2013. 2013.

[61] Vanleeuw L. GeneXpert: An imperfect rollout. Spotlight. 4 Sep 2013.

[62] South African Department of Health. Introduction of new drugs and drug regimens for the management of drug-resistant tuberculosis in South Africa: Policy Framework. 2015 Jun.

[63] WHO. Treatment of tuberculosis guidelines: Fourth edition. WHO Press; 2010. Available from: http://whqlibdoc.who.int/publications/2010/9789241547833_eng.pdf?ua=1

[64] Weyer K. Multidrug-resistant tuberculosis. CME Your SA Journal CPD. 2005;23:74–84. doi: 10.10520/EJC62917

[65] Ndjeka N. Presentation: Multi-Drug Resistant Tuberculosis A Policy Framework on Decentralised and Deinstitutionalised Management in South Africa. In: UCT Lung Institute, editor. Cape Town; 2012.

[66] Geffen N. Anything to Stay Alive: The Challenges of a Campaign for an Experimental Drug. Dev World Bioeth. 2016;16(1):45–54. doi: 10.1111/dewb.12084 25982452

[67] MSF. DR-TB drugs under the microscope: sources and prices for drug-resistant tuberculosis medicines. 2013.

[68] MSF. Factsheet: Why Bedaquiline (TMC207) should be prioritised for drug-resistant TB patients in South Africa. 2012 Oct. Available from: http://www.investor.jnj.com/releasedetail.cfm?releaseid=688371

[69] Conradie F, Meintjes G, Hughes J, Maartens G, Ferreira H, Siwendu S, et al. Clinical access to Bedaquiline Programme for the treatment of drug-resistant tuberculosis. S Afr Med J. 2014;104(3):164–6. doi: 10.7196/samj.7263 24897814

[70] WHO. Experience sharing workshop on the introduction of new drugs for DR-TB treatment in the WHO South East Asia and Western Pacific Regions (24th-25th February 2016). 2016.

[71] Bouton Id TC, De Vos M, Ragan EJ, White LF, Van Zyl L, Theron D, et al. Switching to bedaquiline for treatment of rifampicin-resistant tuberculosis in South Africa: A retrospective cohort analysis. 2019 [cited 18 Jan 2024]. doi: 10.1371/journal.pone.0223308 PMC6797261 31622366

[72] WHO. Automated real-time nucleic acid amplification technology for rapid and simultaneous detection of tuberculosis and rifampicin resistance: Xpert MTB/RIF system: policy statement. World Health Organization; 2011. 26158191

[73] WHO. Global Tuberculosis Report 2015. World Health Organization; 2015.

[74] Diacon AH, Pym A, Grobusch M, Patientia R, Rustomjee R, Page-Shipp L, et al. The diarylquinoline TMC207 for multidrug-resistant tuberculosis. N Engl J Med. 2009;360(23):2397–405. doi: 10.1056/NEJMoa0808427 19494215

[75] MSF. Linezolid fact sheet background: DR-TB in South Africa. 2014.

[76] de Wet P. Motsoaledi: Big pharma’s ‘satanic’ plot is genocide. In: Mail & Guardian. 16 Jan 2014.

[77] MSF. South Africa’s New IP Policy Welcomed by MSF and Fix the Patent Laws Campaign. 31 May 2018.

[78] Harling G, Ehrlich R, Myer L. The social epidemiology of tuberculosis in South Africa: a multilevel analysis. Soc Sci Med. 2008;66(2):492–505. doi: 10.1016/j.socscimed.2007.08.026 17920743

[79] Farmer P. Infections and inequalities: The modern plagues. Berkeley: University of California Press; 1999.

[80] Dixon J, Tameris M. A disease beyond reach: nurse perspectives on the past and present of tuberculosis control in South Africa. Anthropol Southern Africa. 2018;41(4):257–69. doi: 10.1080/23323256.2018.1526096

[81] Abney K. “Containing” tuberculosis, perpetuating stigma: the materiality of N95 respirator masks. Anthropol Southern Africa. 2018;41(4):270–83. doi: 10.1080/23323256.2018.1507675

[82] Compion S. Tuberculosis discourse in South Africa: a case study. University of Pretoria; 2007.

[83] Broom A, Kenny K, Prainsack B, Broom J. Antimicrobial resistance as a problem of values? Views from three continents. Critical Public Health. 2020;31(4):451–63. doi: 10.1080/09581596.2020.1725444

[84] Cox JA, Vlieghe E, Mendelson M, Wertheim H, Ndegwa L, Villegas MV, et al. Antibiotic stewardship in low- and middle-income countries: the same but different? Clin Microbiol Infect. 2017;23(11):812–8. doi: 10.1016/j.cmi.2017.07.010 28712667

[85] Wilkinson A, Ebata A, Macgregor H. Interventions to reduce antibiotic prescribing in LMICs: A scoping review of evidence from human and animal health systems. Antibiotics. MDPI AG; 2019. doi: 10.3390/antibiotics8010002 PMC6466578 30583566

[86] Rodrigues CF. Self-medication with antibiotics in Maputo, Mozambique: practices, rationales and relationships. Palgrave Commun. 2020;6(1). doi: 10.1057/s41599-019-0385-8

[87] Chandler CIR, Hutchinson E. Addressing Antimicrobial Resistance through Social Theory: An Anthropologically Oriented Report. 2016.

[88] Kirby T. Landmark legal ruling sees Indian girl prescribed bedaquiline for XDR-TB. Lancet Respir Med. 2017;5(4):249. doi: 10.1016/S2213-2600(17)30042-5 28169199

[89] WHO. The use of bedaquiline in the treatment of multidrug-resistant tuberculosis: interim policy guidance. 2015. 23967502

[90] Furin J, Brigden G, Lessem E, Rich M, Vaughan L, Lynch S. Global Progress and Challenges in Implementing New Medications for Treating Multidrug-Resistant Tuberculosis. Emerg Infect Dis. 2016;22(3):e151430. doi: 10.3201/eid2203.151430 26885674 PMC4766879

[91] Central TB Division. TB India 2016: Revised National TB Control Programme. 2016.

[92] Dhinwa M, Jha N, Jyani S, Chandra R, Kumar S, Lachyan A, et al. Journey of tuberculosis control, conceptual changes and implications of the shift from NTP to RNTCP to NTEP: A Review. Int J Health Sci (Qassim). 2022;6: 12269–12281. doi: 10.53730/ijhs.v6ns1.8072

[93] Karnan A, Jadhav U, Ghewade B, Ledwani A, Shivashankar P. A Comprehensive Review on Long vs. Short Regimens in Multidrug-Resistant Tuberculosis (MDR-TB) Under Programmatic Management of Drug-Resistant Tuberculosis (PMDT). Cureus. 2024;16(1):e52706. doi: 10.7759/cureus.52706 38384625 PMC10879947

[94] WHO. WHO consolidated guidelines on tuberculosis Module 4: Treatment drug-resistant tuberculosis treatment 2022 update. Geneva: World Health Organization; 2022. 36630546

[95] Thompson AC, Chandler CIR. Addressing antibiotic use: insights from social science around the world. A report collated with social scientists of the Antimicrobials in Society Hub. London; 2021. doi: 10.17037/PUBS.04659562

[96] Derendinger B, Dippenaar A, de Vos M, Huo S, Alberts R, Tadokera R, et al. Bedaquiline resistance in patients with drug-resistant tuberculosis in Cape Town, South Africa: a retrospective longitudinal cohort study. Lancet Microbe. 2023;4(12):e972–82. doi: 10.1016/S2666-5247(23)00172-6 37931638 PMC10842724

[97] Prince RJ, Marsland R. Making and Unmaking Public Health in Africa: Ethnographic and Historical Perspectives. Ohio University Press; 2013. Available from: https://books.google.co.uk/books?id=UvnonQEACAAJ

